# LDLR^–/–^ApoB^100/100^ mice with insulin-like growth factor II overexpression reveal a novel form of retinopathy with photoreceptor atrophy and altered morphology of the retina

**Published:** 2013-08-04

**Authors:** Kati Kinnunen, Suvi E. Heinonen, Giedrius Kalesnykas, Svetlana Laidinen, Hannele Uusitalo-Järvinen, Hannu Uusitalo, Seppo Ylä-Herttuala

**Affiliations:** 1 Department of Biotechnology and Molecular Medicine, A.I. Virtanen Institute for Molecular Sciences, University of Eastern Finland, Kuopio, Finland; 2 Department of Ophthalmology, University of Eastern Finland, Kuopio, Finland; 3 Department of Ophthalmology, Tampere University Hospital, Tampere, Finland; 4 Gene Therapy Unit, Kuopio University Hospital, Kuopio, Finland; 5 Research Unit, Kuopio University Hospital, Kuopio, Finland

## Abstract

**Purpose:**

The aim of this study was to characterize the ocular morphology of low-density lipoprotein receptor–deficient apolipoprotein B-100-only mice, where overexpression of insulin-like growth factor II (IGF-II) has been shown to induce glucose intolerance and increase atherosclerotic lesion progression and calcification.

**Methods:**

Fifteen-month-old mice were examined on a normal chow diet and after 3 months of a high-fat Western diet. IGF-II-negative LDLR^–/–^ApoB^100/100^ littermates and C57Bl/6J mice served as controls. In vivo color images of the fundi were obtained, and eyes were processed either for retinal flat mounts for assessment of neovascularization or for paraffin-embedded samples for immunohistochemical analyses.

**Results:**

IGF-II overexpression and the resulting prediabetic phenotype did not induce microvascular damage when assessed in fundus photographs and retinal whole mounts, and the number of capillaries in IGF-II/LDLR^–/–^ApoB^100/100^ mice was not significantly different from LDLR^–/–^ApoB^100/100^ mice. However, morphology of the inner nuclear, outer plexiform, and outer nuclear layers was altered in the IGF-II/LDLR^–/–^ApoB^100/100^ mice. Moreover, photoreceptor atrophy and thinning of the outer nuclear layer were present. Caspase-3 staining was positive in the photoreceptor inner segment. In addition, retinas of the IGF-II/LDLR^–/–^ApoB^100/100^ mice displayed reduced rhodopsin positivity, consistent with the decreased number of photoreceptor cells.

**Conclusions:**

This study reports a novel form of retinopathy with photoreceptor atrophy and abundant changes in retinal morphology in a mouse model of prediabetes and atherosclerosis.

## Introduction

Type 2 diabetes (T2D) is associated with multiple metabolic abnormalities promoting vascular complications—elevated blood glucose, insulin resistance, and atherogenic lipid abnormalities. This high prevalence of concomitant cardiovascular risk factors contributes to accelerated atherosclerosis and dysfunction of both small and large arteries. As a result, atherosclerotic macrovascular disease processes are more aggressive and diffuse in diabetic patients. Since many of these metabolic perturbations are present well before overt hyperglycemia, processes contributing to vascular complications can be initiated 5–10 years before the development of full-blown diabetes [1].

In addition to macrovascular diseases, which are the major cause of death in diabetes, microvascular complications underlie significant morbidity and premature mortality in diabetic patients. Diabetic retinopathy (DR) is the most common microvascular complication and the leading cause of blindness in the working population in the Western world [2]. The pathogenesis of DR is complex, consisting of both biochemical and histological alterations that lead to damage in retinal vasculature and neural cells. Although it is well known that hyperglycemia is a major risk factor for DR, there is no glycemic threshold for the development of these long-term complications [3]. Therefore, in 25% of T2D patients these changes can be asymptomatically present at the time of diabetes diagnosis [4]. In addition, while generally associated with macrovascular complications, dyslipidemia has been shown to correlate with the status of DR; increased levels of triglycerides and low-density lipoproteins (LDL) in plasma as well as a concentration of LDL particles and apolipoprotein B (ApoB) in the retinal hard exudates have been associated with the severity of retinopathy [5,6].

Metabolic disorders often associated with T2D predispose the individual to other eye diseases. Age-related macular degeneration (AMD), the leading cause of irreversible blindness among older people [7], is initiated with the accumulation of lipid-rich deposits under the RPE and within Bruch’s membrane [8,9]. Epidemiologic studies have suggested that dietary fat and cholesterol play an important role in the pathogenesis of AMD [10]. The role of LDL cholesterol in the pathogenesis of AMD is also seen in LDL receptor knockout (LDLR^–/–^) mice, which exhibit abnormalities in Bruch’s membrane characterized by thickening and deposition of lipid-rich particles [11] similar to the drusen seen in patients with AMD [12]. ApoB is also known to be present in RPE and Bruch’s membrane under normal conditions and in drusen and basal deposits of AMD patients [13]. Similarly, transgenic mice expressing human ApoB develop basal laminar deposits in Bruch’s membrane after a high-fat diet [14]. Moreover, when made hypercholesterolemic in the LDLR^–/–^ background, these human ApoB transgenic LDLR^–/–^ mice have been found to accumulate esterified cholesterol at the basement of the RPE and develop alterations seen in electroretinography, indicating photoreceptor dysfunction [15].

We previously generated a novel mouse model of the prediabetic features and atherosclerosis by overexpressing insulin-like growth factor II (IGF-II) in an atherogenic LDLR^–/–^ApoB^100/100^ background and characterized the macrovascular phenotype [16,17]. We found that in old animals, disturbances in glucose metabolism increase lesion progression and calcification without changes in plasma lipid levels compared to only hypercholesterolemic LDLR^–/–^ApoB^100/100^ controls. In this study, we characterized the ocular morphology of the IGF-II/LDLR^–/–^ApoB^100/100^ mouse model.

## Methods

### Animals

IGF-II/LDLR^–/–^ApoB^100/100^ mice represent a model of diabetic macroangiopathy and have been previously described [16]. In brief, mice deficient of LDL receptor and expressing only apolipoprotein B100 (LDLR^–/–^ApoB^100/100^) [18] in a C57BL/6×129/SvJae background (The Jackson Laboratory, Bar Harbor, ME) were crossbred with C57BL6/SJL mice overexpressing IGF-II in pancreatic β-cells [19]. IGF-II-negative LDLR^–/–^ApoB^100/100^ littermates served as controls. Both sexes were included in the study groups. In principle, 15-month-old mice that had either been fed ad libitum a normal chow diet (R36; Lactamin, Kimsta, Sweden, n=6/strain) or fed a high-fat Western diet for 12–15 months (TD 88,173, Harlan Teklad, Madison, WI, 42% of calories from fat and 0.15% from cholesterol, no sodium cholate, n=9–12/strain) were examined. We also included 15-month-old C57Bl/6J mice on a Western diet (n=9) in histological analyses. During experiments mice were anesthetized using xylazine (10 mg/kg) and ketamine (80 mg/kg) subcutaneously and euthanized using carbon dioxide. Mice were housed in groups and maintained in a temperature- and humidity-controlled environment with a 12-h light–dark cycle at the National Laboratory Animal Centre in Kuopio. All protocols involving the use of mice adhered to the regulations set forth in the Association for Research in Vision and Ophthalmology (ARVO) Statement for the Use of Animals in Ophthalmic and Vision Research and were approved by the Experimental Animal Committee of the University of Kuopio.

### Metabolic analyses

Blood glucose was determined using a Glucometer Elite analyzer (Bayer, Elkhart, IN). Plasma insulin levels were measured using an ELISA kit (Rat/mouse Insulin ELISA Kit; Linco Research Inc., St. Charles, MO). From overnight fasted plasma samples, triglycerides (Ecoline® S+, Diasys, Diagnostic Systems Gmbh, Holzheim, Germany) and total cholesterol (Ecoline® 25, Merck KgaA, Darmstadt, Germany) were measured as described previously [20].

### Fundus photography

In vivo color images of the fundi were obtained by fundus camera (Nikon D70s; Nikon Corp., Tokyo, Japan). Photographs were also taken from the anterior segments. In addition, the eyes were evaluated by a slit lamp and ophthalmoscopy. The fundi were photographed after dilation of the pupils with one drop of tropicamide (5 mg/ml) and phenylephrine hydrochloride (100 mg/ml; Kuopio University Hospital, Kuopio, Finland).

### Retinal whole mounts

Mice were euthanized and the eyes enucleated. After a 10-min fixation in 4% paraformaldehyde (PFA), the cornea, lens, sclera, and vitreous were excised and the retina isolated. Retina flat mounts (from four to five 15-month-old animals/group on a Western diet) were fixed with 4% PFA for 1 h and stained for capillaries with fluorescence-conjugated isolectin *Griffonia simplicifolia* (1:150, GS lectin, I-21413; Molecular Probes, Eugene, OR). For immunohistochemistry, retinas were blocked in 20% fetal bovine and 20% normal goat serum and stained for pericytes with anti-chondroitin sulfate proteoglycan antibody, a cell-surface proteoglycan marker (1:200, Chemicon, Billerica, MA), followed by appropriate fluorescence-conjugated secondary antibodies. Whole mount retinas were imaged using confocal microscopy (Olympus IX70, Perkin Elmer, Waltham, MA) with a 4×objective lens. Overlapping images were assembled into whole retina montages using Photoshop 6.0 (Adobe, San Jose, CA). Areas of vascular obliteration and neovascular tufts were quantified from these montages.

### Histological evaluation of paraffin-embedded retinal sections

Mice were sacrificed and perfused with PBS (137 mM sodium chloride, 10 mM phosphate, 2.7 mM potassium chloride) and 4% PFA, pH 7.4. Immunohistochemical stainings were performed on serial 6-μm sections. Morphological alterations were analyzed with hematoxylin and eosin (H&E)-stained radial sections. Samples were immunostained with antibodies against IGF-II (1:100; GroPep Limited, Thebarton, Australia), macrophages (mMQ AIA31240, 1:5,000; Accurate Chemical & Scientific Corp., Westbury, NY), CD31 (platelet endothelial cell adhesion molecule [PECAM]- 1,1:50; BD Biosciences PharMingen, San Diego, CA), caspase-3 (1:250, Promega Corp., Fitchburg, WI), receptor for advanced glycation end products (RAGE, 1:50; R&D Systems, Minneapolis, MN), interleukin 6 (IL-6, 1:300; Lifespan Biosciences, Seattle, WA), intercellular adhesion molecule 1 (ICAM-1, 1:500; R&D Systems, Minneapolis, MN and vascular cell adhesion molecule 1 (VCAM- 1), 1:100; Chemicon), caspase-1 (1:100; Imgenex, Odisha, India), calbindin (1:1,000; SWANT, Bellinoza, Switzerland), calretinin (1:1,000; SWANT), rhodopsin (1:500, Chemicon), and heat shock proteins 25 kDa (HSP-25, 1:500), 60 kDa (HSP-60, 1:500), and 70 kDa (HSP-70,1:200; all from Stressgenbioreagents, Kampenhout, Belgium). Terminal deoxynucleotidyl transferase dUTP nick end labeling (TUNEL; Apop Tag plus peroxidase in situ apoptosis detection kit; Millipore, Billerica, MA) was used to demonstrate apoptosic cells in the retina. Avidin-biotin–horseradish peroxidase system with 3,3′-Diaminobenzidine (DAB) was used for immunostainings. Photographs of the histological sections were taken using an Olympus AX70 microscope (Olympus Optical, Tokyo, Japan) and analyzed using analysis software (Soft Imaging System Gmbh, Lakewood, CO).

The number of cells in the inner (INL) and outer nuclear layers (ONL) and ganglion cell layer (GCL) was analyzed from H&E stained sections from the posterior pole near the optic nerve head in five animals per group and expressed as a percentage compared to that in the C57Bl/6J control group. Capillary density (capillaries/mm^2^) and mean capillary area (mm^2^) in the retina were calculated from CD31-immunostained sections. Quantification was performed from three sections per mouse, and three fields at 400× magnification were randomly selected in each section and analyzed with analySIS software (Soft Imaging System Gmbh).

### Statistical analyses

An independent samples *t* test was used for metabolic analyses to evaluate statistical significance. One-way ANOVA with Bonferroni’s post test was used when analyzing cell counts in retinal layers. Numerical values for each measurement are shown as mean±standard deviation or mean±standard error of the mean. All statistical analyses were performed using GraphPad Prism version 4.0 (GraphPad Software, San Diego, CA).

## Results

### Metabolic parameters

Metabolic parameters for the 15-month-old animals are presented in Table 1. On a Western diet, the IGF-II/LDLR^–/–^ApoB^100/100^ mice showed increased fasting glucose levels compared to the LDLR^–/–^ApoB^100/100^ controls (6.0±2.1 versus 3.8±1.6 mmol/l, p<0.05). No significant differences between the groups in bodyweights were detected for either the normal or Western diet. Profound hypercholesterolemia developed with the Western diet both in IGF-II/LDLR^–/–^ApoB^100/100^ and LDLR^–/–^ApoB^100/100^ control mice without any differences between the groups. No changes were present in fasting triglycerides.

**Table 1:** Metabolic parametres of 15-month-old IGF-II/LDLR^−/−^ApoB^100/100^ mice and LDLR^−/−^ApoB^100/100^ controls on normal and Western diet.

Weight (g)	Normal diet		Western diet	
	LDLR^–/–^ApoB^100/100^	IGF-II/ LDLR^–/–^ApoB^100/100^	LDLR^–/–^ApoB^100/100^	IGF-II/ LDLR^–/–^ApoB^100/100^
Male	30.0±4.2	31.0±4.5	40.5±5.3	40.4±3.3
Female	26.2±2.9	29.2±4.1	31.0±3.5	32.1±3.1
Glucose (mmol/l)	3.8±0.9	4.7±1.6	3.8±1.6	6.0±2.1*
Insulin (ng/ml)	1.1±0.3	1.3±0.4	0.6±0.03	0.7±0.02
Triglycerides (mmol/l)	1.5±0.7	1.7±0.9	1.4±0.4	1.9±1.0
Cholesterol (mmol/l)	8.3±1.2	7.6±1.8	22.7±4.5	21.2±6.0

Values are mean ±SD or mean±SEM (insulin) and represent values after overnight fasting. *p<0.05 compared to the LDLR^−/−^ApoB^100/100^ controls. Number of animals analyzed is 7–12.

### In vivo examination of the eyes

In vivo examination of the eyes by biomicroscopy revealed no significant changes in either diabetic IGF-II/LDLR^–/–^ApoB^100/100^ mice fed with a normal or Western diet or in control animals. The pupils dilated normally with mydriatics indicating normal anatomy and physiology of the anterior parts of the eye. Signs of hyperpermeability of the vessels in the retinal layers, such as hemorrhages, edema, or exudates, were not observed. Retinal photographs revealed no clinically relevant changes in the retinas either in the IGF-II/LDLR^–/–^ApoB^100/100^ or control mice (data not shown).

### Diabetes did not induce vascular changes in the retina

**Figure 1:**
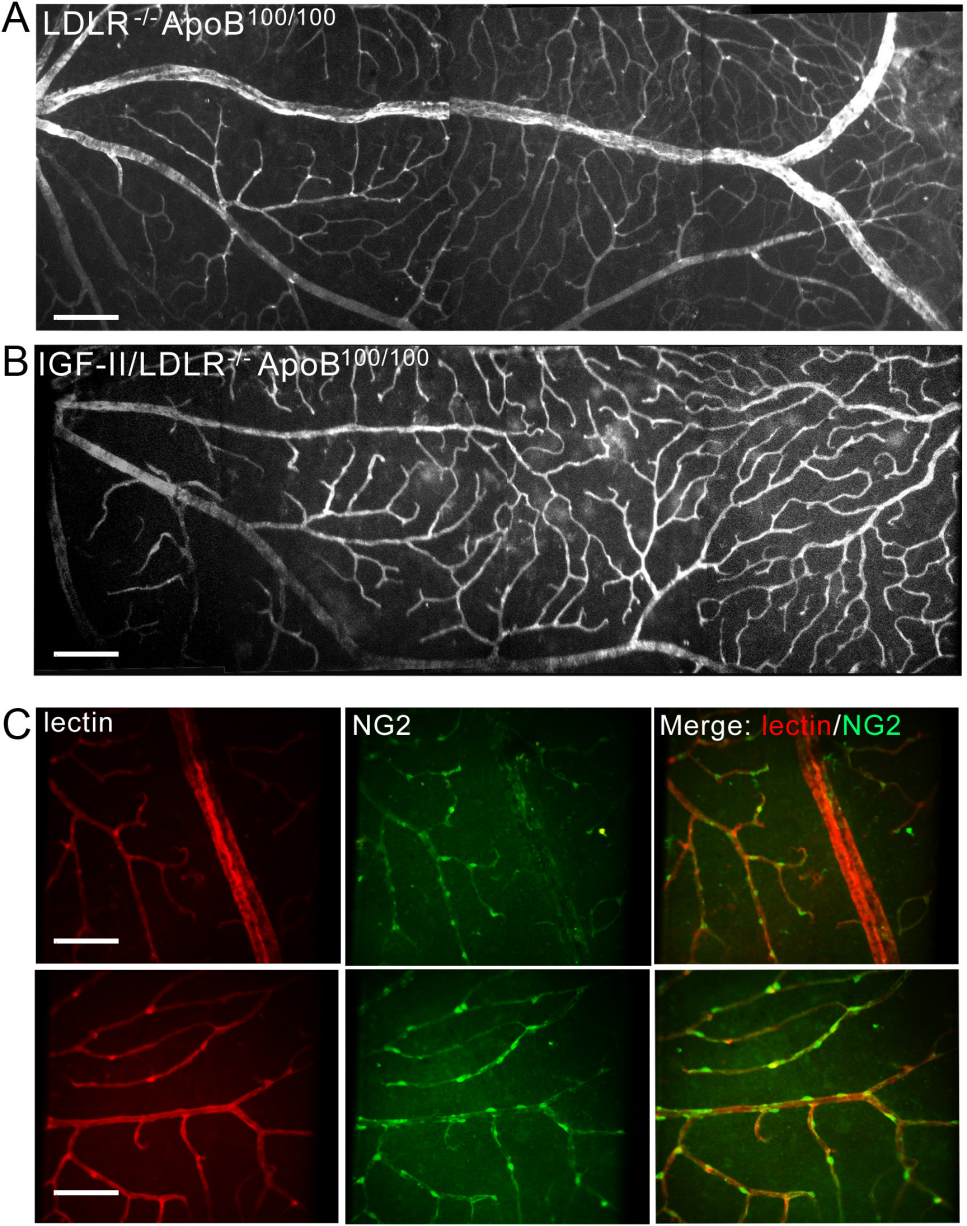
No neovessel formation was observed in low-density lipoprotein receptor–deficient apolipoprotein B-100-only mice overexpressing insulin-like growth factor II (IGF-II/LDLR^–/–^ApoB^100/100^). Retinal whole mounts reveal a normal retinal vasculature in both 15-month-old IGF-II/LDLR^–/–^ ApoB^100/100^ mice and LDLR^–/–^ApoB^100/100^ mice (**A** and **B**) fed with a normal diet. Lectin staining of capillaries (**C**, left panel), Chondroitin Sulfate Proteoglycan (NG2) staining for pericytes (**C**, middle panel), and a merged image (**C**, right panel) of control LDLR^–/–^ApoB^100/100^ (**C**, upper panel) and IGF-II/LDLR^–/–^ApoB^100/100^ mice (**C**, lower panel) reveal a normal retinal vasculature in both 15-month-old IGF-II/LDLR^–/–^ApoB^100/100^ mice and LDLR^–/–^ApoB^100/100^ mice (**A** and **B**) fed with a normal diet. The scale bar is 50 µm in **A** and **B**, and 25 µm in **C**.

Diabetes in the IGF-II/LDLR^–/–^ApoB^100/100^ mice did not induce retinal neovessel formation on normal chow (Figure 1) or a Western diet (data not shown). No early microvascular diabetic changes, such as micro-aneurysms, hemorrhages, intraretinal microvascular abnormalities or lipid exudates were found in the retinas of IGF-II/LDLR^–/–^ApoB^100/100^ or control mice.

### Histopathological changes and retinal degeneration in diabetic mice

**Figure 2:**
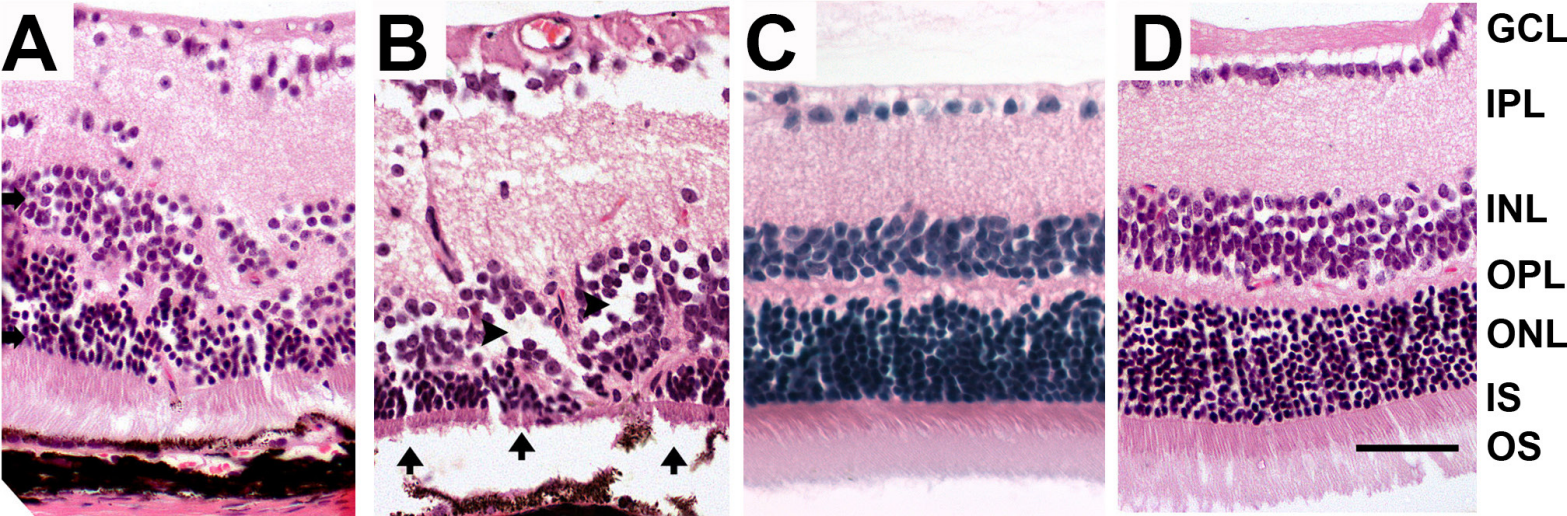
Histopathological changes in the retina. **A**: Hematoxylin and eosin-stained radial sections show morphological alterations with displaced cells (arrows) in 15-month-old low-density lipoprotein receptor–deficient apolipoprotein B-100-only mice overexpressing insulin-like growth factor II (IGF-II/LDLR^–/–^ApoB^100/100^). **B**: Photoreceptor atrophy (arrows) and thinning of the outer nuclear layer (arrowheads) in outer nuclear layer are also present. **C**, **D**: Normal retinal morphology in 15-month-old C57Bl/6J (**C**) and a nondiabetic LDLR^–/–^ApoB^100/100^ mouse (**D**). Original magnification in the figure is 400×, and scale bar is 25 μm. Definitions for the abbreviations are: GCL, ganglion cell layer; IPL, inner plexiform layer; INL, inner nuclear layer; OPL, outer plexiform layer; ONL, outer nuclear layer; IS, inner segment; OS, outer segment.

In H&E-stained paraffin sections, altered retinal morphology was observed in  IGF-II/LDLR^–/–^ApoB^100/100^ mice. Changes were present in all (six of six) old mice on a normal diet and in six of nine (67%) mice on a Western diet. There were displaced cells in the inner plexiform layer (IPL), and the morphology of the INL, outer plexiform layer (OPL), and ONL was abundantly altered. Photoreceptor atrophy was also observed in the IGF-II/LDLR^–/–^ApoB^100/100^ mice (Figure 2).The LDLR^–/–^ApoB^100/100^ mice presented normal retinal morphology similar to C57Bl/6J controls.

**Figure 3:**
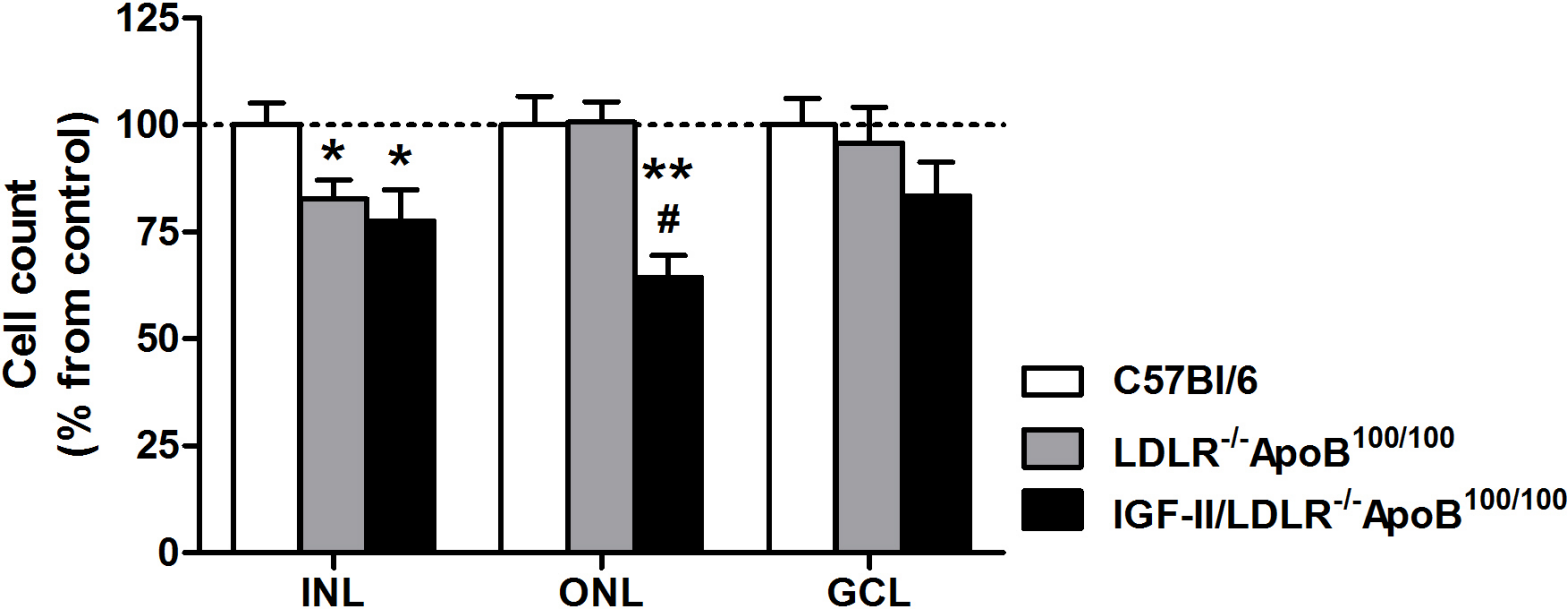
Evaluation of the cell number in the retinal layers in 15-month-old mice. Low-density lipoprotein receptor–deficient apolipoprotein B-100-only mice overexpressing insulin-like growth factor II (IGF-II/LDLR^–/–^ApoB^100/100^) had a statistically significant decrease in the inner nuclear layer (INL) cell count compared to the C57Bl/6J control group (expressed as a percentage of cell counts in the C57Bl/6J mice). The outer nuclear layer (ONL) cell number was also significantly decreased in IGF-II/LDLR^–/–^ApoB^100/100^ mice compared to both C57Bl/6J and LDLR^–/–^ApoB^100/100^ mice. No significant differences were seen in the number of cells in the ganglion cell layer (GCL). * denotes p value <0.05 versus C57Bl/6J mice; ** denotes p value <0.01 versus C57Bl/6 mice; # denotes p value <0.01 versus LDLR^–/–^ApoB^100/100^ mice. Values denote mean ± standard error of the mean of five mice in each group.

**Figure 4:**
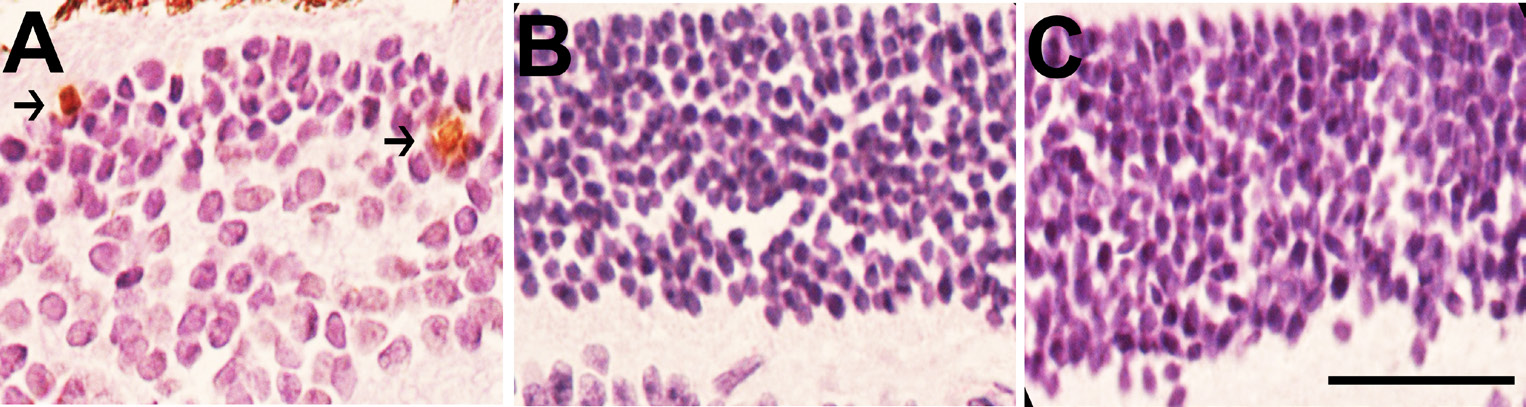
Apoptotic cells in the retinal layers of 15-month-old mice. Terminal deoxynucleotidyl transferase dUTP nick end labeling (TUNEL) showed apoptotic cells (arrows) in the outer nuclear layer (ONL) of low-density lipoprotein receptor–deficient apolipoprotein B-100-only mice overexpressing insulin-like growth factor II (IGF-II/LDLR^–/–^ApoB^100/100^). **A**: No positivity was found in the LDLR^–/–^ApoB^100/100^ mice (**B**) or C57Bl/6J mice (**C**). Original magnification in the figure is 400×, and the scale bar is 25 μm.

Cellularity in the ONL of IGF-II/LDLR^–/–^ApoB^100/100^ mice on a normal diet was decreased compared to both LDLR^–/–^ApoB^100/100^ mice (p<0.01) and C57Bl/6J controls (p<0.01; Figure 3). In addition, apoptotic cells indicated by TUNEL staining were present in the ONL in 63% of IGF-II/LDLR^–/–^ApoB^100/100^ mice, while no positivity was seen in LDLR^–/–^ApoB^100/100^ or C57Bl/6J mice (Figure 4).The number of cells in the INL in both IGF-II/LDLR^–/–^ApoB^100/100^ mice and LDLR^–/^–ApoB^100/100^ mice was decreased compared to C57Bl/6J controls (Figure 3), but the difference was statistically significant only in IGF-II/LDLR^–/–^ApoB^100/100^ mice (p<0.05). There were no statistically significant differences in the number of cells in the GCL between the groups, but a trend toward a reduced cell count was seen in IGF-II/LDLR^–/–^ApoB^100/100^ mice.

**Figure 5:**
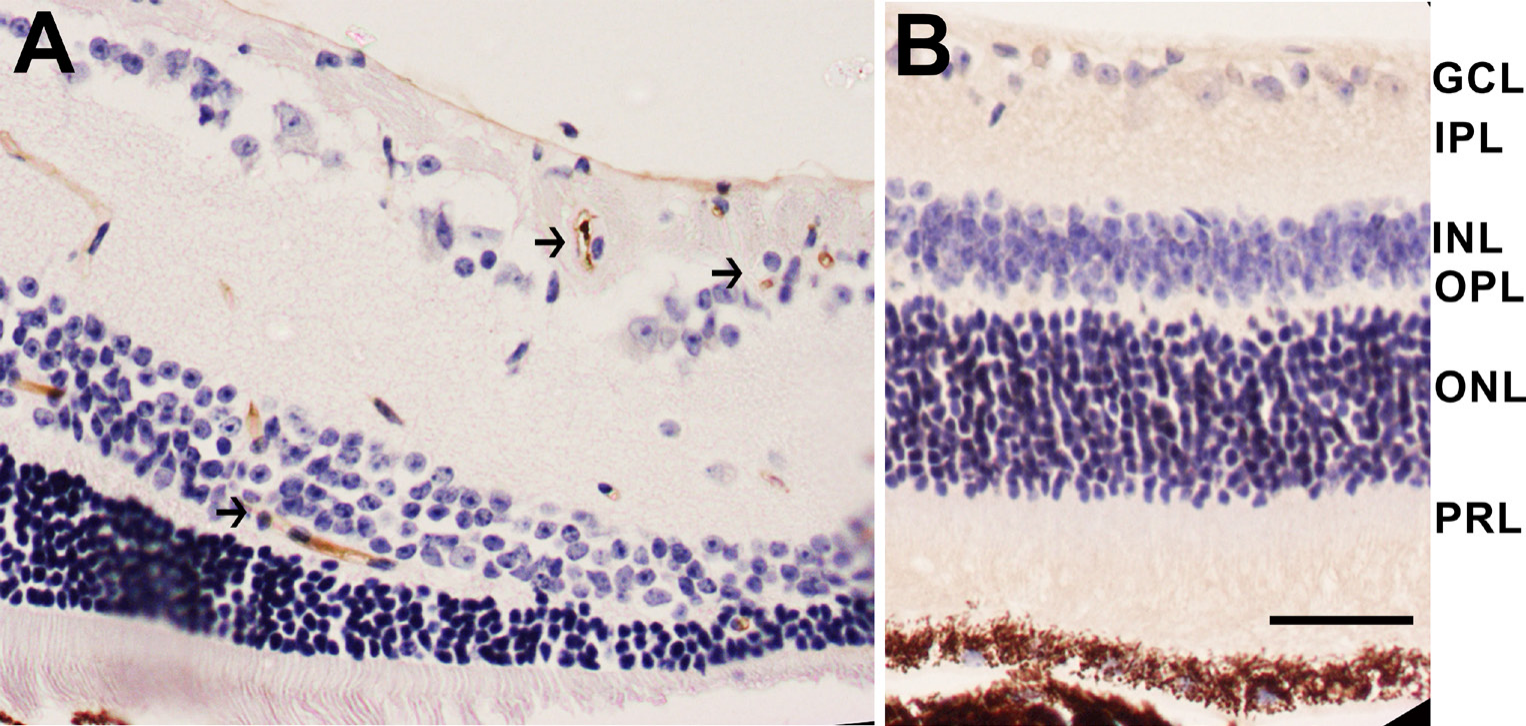
Immunohistochemical analysis of 15-month old mice. **A**, **B**: Increased immunoreactivity for insulin-like growth factor II (IGF-II) is present in the vasculature of the low-density lipoprotein receptor–deficient apolipoprotein B-100-only mice overexpressing IGF-II (IGF-II/LDLR^–/–^ApoB^100/100^; arrows, **A**), while no positivity is seen in the LDLR^–/–^ApoB^100/100^ control (**B**). Original magnification in the figure is 400×, and the scale bar 25 μm. Definitions for the abbreviations are: GCL, ganglion cell layer; IPL, inner plexiform layer; INL, inner nuclear layer; OPL, outer plexiform layer; ONL, outer nuclear layer; PRL, photoreceptor layer.

**Figure 6:**
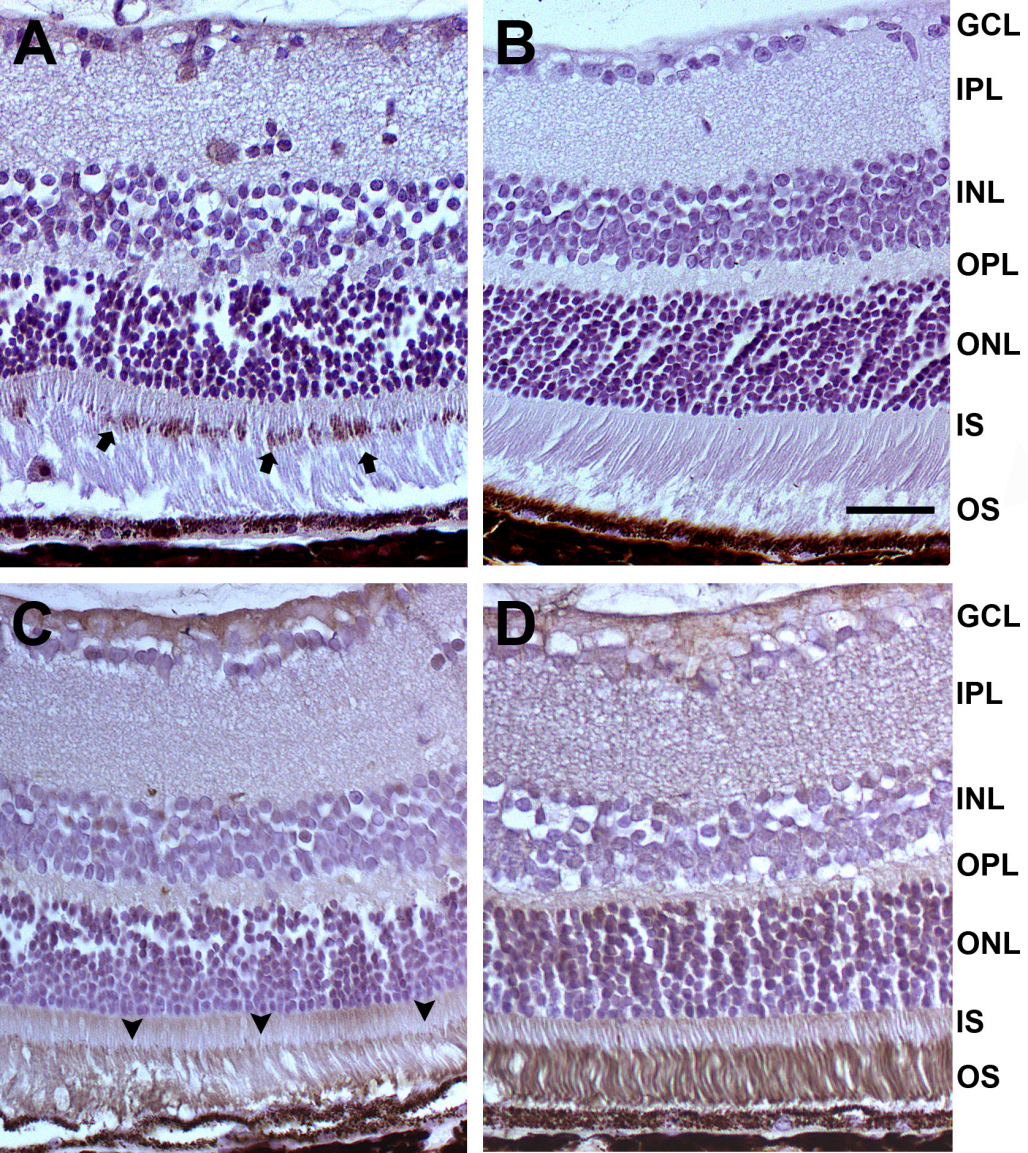
Immunohistochemical analysis of 15-month old mice. **A**, **B**: Increased immunoreactivity for caspase-3 is present in the inner photoreceptor segment of a low-density lipoprotein receptor–deficient apolipoprotein B-100-only mouse overexpressing insulin-like growth factor II (IGF-II/LDLR^–/–^ApoB^100/100^; arrows, **A**), while no positivity is seen in the LDLR^–/–^ApoB^100/100^ control (**B**). **C**, **D**: Reduced rhodopsin staining is observed in the retinas of IGF-II/LDLR^–/–^ApoB^100/100^ mice, consistent with the decreased number of photoreceptor cells (arrowheads, **C**), whereas intense staining was present in the nondiabetic LDLR^–/–^ApoB^100/100^ mice (**D**). Original magnification in the figure is x400, and the scale bar is 25 μm. Definitions for the abbreviations are: GCL, ganglion cell layer; IPL, inner plexiform layer; INL, inner nuclear layer; OPL, outer plexiform layer; ONL, outer nuclear layer; IS, inner segment; OS, outer segment.

In immunohistochemical analyses, IGF-II was found to be expressed in the capillaries of the IGF-II/LDLR^–/–^ApoB^100/100^ mice but not in the control mice (Figure 5). However, the number of capillaries in the IGF-II/LDLR^–/–^ApoB^100/100^ mice was not increased compared to control mice (data not shown). Capillaries were similarly situated in the nerve fiber layer and in the OPL. Displaced cells in the IPL stained positively with calbindin and calretinin, which are specific markers for amacrine cells. There were no differences between the control and diabetic mice in retinal expression of HSPs. Moreover, the retinal expression of RAGE, caspase-1, IL-6, and adhesion molecules ICAM-1 and VCAM-1 were similar between the groups (data not shown). However, caspase-3 was abundantly positive in the inner segment of the photoreceptor cells in IGF-II/LDLR^–/–^ApoB^100/100^ mice (Figure 6). In addition, apoptotic cells were found in the ONL layer, suggestive of damages in the photoreceptor nuclei. No such staining was observed in the LDLR^–/–^ApoB^100/100^ control littermates. To evaluate the retinal structure, rhodopsin immunostaining was performed. Rhodopsin staining was reduced in the retinas of IGF-II/LDLR^–/–^ApoB^100/100^ mice, consistent with the decreased number of photoreceptor cells (Figure 6).

## Discussion

In the present study the ocular morphology of IGF-II/LDLR^–/–^ApoB^100/100^ mice, demonstrating a prediabetic state in a hypercholesterolemic background, was characterized. Compared to hyperlipidemic LDLR^–/–^ApoB^100/100^ controls, we found that IGF-II/LDLR^–/–^ApoB^100/100^ mice on a control or Western diet displayed altered retinal morphology and photoreceptor atrophy, suggesting the presence of retinal degeneration and a novel form of retinopathy.

Several factors could potentially contribute to the observed ocular changes. First, hyperglycemia is known to trigger a cascade of metabolic and biochemical changes in the retina long before any associated disease can be detected [21]. Moreover, it is associated with biochemical alterations and apoptosis of neuronal and vascular cells in the retina [22,23]. Many diabetic mouse models, such as leptin receptor-deficient db/db mice [24] and mice where hyperglycemia is induced by streptozocin [25] or galactose feeding [26], develop vascular and neural changes typical of early stages of DR. However, overexpression of IGF-II in the LDLR^–/–^ApoB^100/100^ background results in impaired glucose tolerance, insulin resistance, and moderately increased blood glucose levels [16], which together simulate more of a metabolic disorder or a prediabetic state rather than full-blown diabetes. Although the absence of profound hyperglycemia could contribute to the observed lack of typical diabetic vascular alterations in this model, it is not exclusionary since the development of vascular lesions does occur in type 2 diabetic patients who already have a moderate degree of hyperglycemia [27].

Second, disturbances in glucose metabolism in the LDLR^–/–^ApoB^100/100^ mice were caused by overexpression of IGF-II, and thus the possibility of direct effects of IGF-II on the eye should also be considered. In general, many growth factors have been associated with the ocular changes seen in diabetes and more specifically with the development of vascular lesions in DR. Indeed, growth factors have been needed to achieve the most advanced DR phenotypes in mice where severe hyperglycemia alone has not been enough to elicit profound DR [28]. One such example model is the Akimba mouse where transgenic upregulation of vascular endothelial growth factor (VEGF) in photoreceptors induces neovascular changes and characteristics of proliferative DR [29]. Also, transgenic mice with a robust overexpression of IGF-I in the retina display most of the alterations seen in human DR [30], while a similar model with a lower expression level of IGF-I does not develop any ocular abnormalities [31]. However, the role of IGF-II has been studied to a lesser extent. IGF-II is an important growth factor during embryonic development, but the levels decrease considerably after birth and are very low in adult rodents [32]. Despite the low systemic levels, IGF-II has been observed to be present in the retina of adult rats [33]. This was not noted in C57BL/6 or LDLR^–/–^ApoB^100/100^ control animals in the present study. However, IGF-II expression was observed in the retinal capillaries of the IGF-II transgenic mice. In these mice, the transgene expression is regulated by the rat insulin-I promoter (RIP-I), leading to expression mainly in pancreatic β-cells, while no expression in liver or muscle cells has been observed [19]. Nevertheless, it seems that the promoter is also active in the retina since the same RIP-I/β-globin expression vector [34] has been used in the generation of IGF-I transgenic mice with strong retinal expression of IGF-I and a profound DR phenotype [29]. In fact, biosynthesis of preproinsulin has been detected in various sites outside the pancreas, e.g., in the rat retina [35], which could explain the observed retinal expression of the IGF-II transgene in the present study.

Could increased IGF-II expression then underlie the observed changes in the retina? Increased ocular IGF-II levels have been observed in mice with oxygen-induced retinopathy [36,37]. In those studies IGF-II was stated to be important for photoreceptor cell survival and vessel growth [37], with its angiogenic properties being mediated through direct effects as well as indirectly through VEGF induction [36]. However, despite the increased expression of IGF-II in the retinal capillaries, the observed abnormalities in our study were the opposite from those stated previously in other models. Therefore, alternative explanations for the observed pathology should be considered.

Another possible factor driving the observed retinal abnormalities is dyslipidemia. This has been shown to contribute to the pathogenesis of different ocular diseases, and accumulation of lipids into the sub-RPE space has been observed in different hypercholesterolemic mouse models [11,15,38,39]. In the present study, the only common finding for both hyperlipidemic strains was the significantly decreased cell number in INL when compared to normolipidemic C57Bl/6J mice. This has also been observed in hypercholesterolemic ApoE-deficient mice, indicating some degree of degeneration of the inner retinal cell layers [40]. Therefore, it is possible that supraphysiological cholesterol levels have also induced such changes in the LDLR^–/–^ApoB^100/100^ background. However, since no other changes in the sub-RPE space typical to hyperlipidemia were encountered and since IGF-II transgenic mice presented significantly more intraretinal changes than the LDLR^–/–^ApoB^100/100^ mice with similar plasma lipid levels, it is unlikely that hyperlipidemia would be the major underlying factor of the observed changes in the IGF-II/LDLR^–/–^ApoB^100/100^ mice.

What about genetic factors? It is generally acknowledged that inbred mouse strains carry mutations that can have an effect on the ocular phenotype. For example, the retinal degeneration 1 mutation *Pde6b^rd1^* is present in many generally used inbred mouse strains and causes blindness when two copies of the mutated gene are present [41]. One of the strains carrying this mutation is SJL, which is also the strain used in the generation of the original IGF-II transgenic mouse [19]. Therefore, in theory, it is possible that the mutation would still be present in the studied animals. However, this is not presumable for two reasons. First, the IGF-I transgenic mice do not show any signs of retinal degeneration although they are in a pure SJL background [29]. Second, after crossbreeding with the LDLR^–/–^ApoB^100/100^ strain, the IGF-II/LDLR^–/–^ApoB^100/100^ mice have been backcrossed for over 10 years to LDLR^–/–^ApoB^100/100^ mice in the C57BL/6×129/SvJae background.

Since chronic inflammation is involved in the pathogenesis of DR [42,43] and since the aortic walls of IGF-II/LDLR^–/–^ApoB^100/100^ mice were found to present a more inflammatory phenotype compared to LDLR^–/–^ApoB^100/100^ mice in earlier studies [16,17], retinas were examined for signs of inflammation. However, the retinal expression of adhesion molecules ICAM-1 and VCAM-1 as well as caspase-1 was not increased in the IGF-II/LDLR^–/–^ApoB^100/100^ mice. Advanced glycation end products have also been suggested to induce retinal expression of IL-6 [44], which subsequently induces the expression of VEGF [45], the primary mediator of retinal neovascularization. Nevertheless, in the present study the expressions of either IL-6 or RAGE were not increased in the IGF-II/LDLR^–/–^ApoB^100/100^ mice, which is in line with the absence of angiogenic changes.

Increased activation of caspase-3 was seen in retinas of the IGF-II/LDLR^–/–^ApoB^100/100^ mice. The number of cells in the ONL, consisting mainly of photoreceptor cell nuclei, was significantly decreased in IGF-II/LDLR^–/–^ApoB^100/100^ mice compared to both LDLR^–/–^ApoB^100/100^ mice and C57Bl/6J control mice. Together with TUNEL-positive cells and reduced rhodopsin positivity, this is indicative of photoreceptor atrophy. During the prediabetic phase, anomalies might be developing due to changes in the circulation and insufficient oxygen supply in the retina [46,47]. Therefore, it has been suggested that the level of photoreceptor metabolism could play a role in the initiation and progression of DR [48,49]. Reduced retinal metabolism would decrease oxygen demand and thus ameliorate the hypoxia that triggers VEGF production and consequent neovascularization. Indeed, it has been observed both in humans and mice that conditions associated with photoreceptor degeneration, such as AMD and retinitis pigmentosa, are protective from reactive retinal neovascularization typical in DR [48,49]. In fact, caspase-3 activation is generally seen in animal models with either inherited or induced photoreceptor degeneration: the rd-1 mouse [50,51], rhodopsin mutant rat [52], chemically induced retinal degeneration [53], and photoreceptor degeneration following blue light exposure [54]. Hence, it is possible that excessive photoreceptor atrophy is also protective against diabetic microvascular changes in the IGF-II/LDLR^–/–^ApoB^100/100^ mice.

In conclusion, IGF-II/LDLR^–/–^ApoB^100/100^ mice displayed photoreceptor atrophy and abundant changes in retinal morphology. As a limitation to the study, only one time point was evaluated, and therefore conclusions about the temporal profile of the morphological changes cannot be drawn. Importantly, the observed changes were not characteristic of typical diabetic retinopathy but describe a novel form of retinopathy. Further studies assessing, e.g., the ocular ultrastructure and retinal function with electroretinography, are needed for elucidating the exact etiology of these pathological changes and for a complete ocular characterization of this animal model.
